# The Late‐Quaternary Extinctions Gave Rise to Functionally Novel Herbivore Assemblages

**DOI:** 10.1002/ece3.71101

**Published:** 2025-03-13

**Authors:** Simon D. Schowanek, Matt Davis, Erick J. Lundgren, Owen Middleton, John Rowan, Daniel Ramp, Christopher J. Sandom, Jens‐Christian Svenning

**Affiliations:** ^1^ Center for Biodiversity Dynamics in a Changing World (BIOCHANGE), Department of Biology Aarhus University Aarhus Denmark; ^2^ Department of Ecology Norwegian University of Life Sciences (NMBU) Ås Norway; ^3^ Natural History Museum of Los Angeles County Los Angeles California USA; ^4^ Center for Ecological Dynamics in a Novel Biosphere (ECONOVO), Department of Biology Aarhus University Aarhus Denmark; ^5^ Centre for Compassionate Conservation, Transdisciplinary School University of Technology Sydney Ultimo Australia; ^6^ School of Life Sciences University of Sussex Sussex UK; ^7^ Department of Archaeology University of Cambridge Cambridge UK

**Keywords:** biome shifts, ecological novelty, functional ecology, large herbivore, megafauna extinctions

## Abstract

Various authors have suggested that extinctions and extirpations of large mammalian herbivores during the last ca. 50,000 years have altered ecological processes. Yet, the degree to which herbivore extinctions have influenced ecosystems has been difficult to assess because past changes in herbivore impact are difficult to measure directly. Here, we indirectly estimated changes in (theorised) herbivore impact by comparing the functional composition of current large (≥ 10 kg) mammalian herbivore assemblages to those of a no‐extinction scenario. As an assemblage's functional composition determines how it interacts with its environment, changes in functional compositions should correspond to changes in ecological impacts. We quantified functional composition using the body mass, diet and life habit of all wild herbivorous mammal species (*n* = 502) present during the last 130,000 years. Next, we assessed whether these changes in functional composition were large enough that the resulting assemblages could be considered functionally novel. Finally, we assessed where novel herbivore assemblages would most likely lead to changes in biome state. We found that 47% of assemblages are functionally novel, indicating fundamental changes in herbivore impacts occurred across much of the planet. On 20% of land, functionally novel herbivore assemblages have arisen in areas where alternative biome states are possible depending on the disturbance regime. Thus, in many regions, the late‐Quaternary extinctions and extirpations altered herbivore assemblages so profoundly that there were likely major consequences for ecosystem functioning.

## Graphical abstract

We find that 47% of assemblages are functionally novel, indicating that fundamental changes in herbivore impacts have occurred across the world. On 20% of land, functionally novel herbivore assemblages arose in areas where alternative biome states are possible depending on the disturbance regime. Thus, in many regions, the late‐Quaternary extinctions and extirpations altered herbivore assemblages so profoundly that there were likely major consequences for ecosystem functioning.

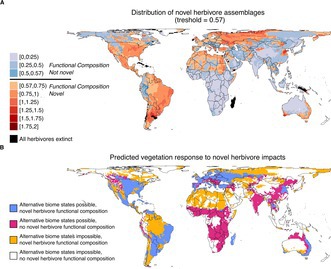

## 1 Introduction

Large herbivores (here terrestrial, mammalian herbivores ≥ 10 kg [Owen‐Smith 2013]) affect ecological processes, such as vegetation dynamics, fire regimes and nutrient fluxes via herbivory and non‐trophic effects (together, herbivore impacts) (Pringle et al. 2023). Large herbivores and their impacts have been ubiquitous in terrestrial ecosystems across the world for at least 50 million years (Janis 2008; Smith et al. 2010), and have been a key driver of ecological processes. However, extinctions and extirpations during the ca. 50,000 years have simplified modern herbivore assemblages globally (Schowanek et al. 2021). Various authors have suggested that this reduction in herbivore abundance and diversity has altered herbivores' impact on ecological processes, and that these reductions may have changed the functioning of ecosystems worldwide (Bakker et al. 2016; Gill 2014; Owen‐Smith 1987; Pedersen et al. 2023; Zimov et al. 1995). Unfortunately, the degree to which global impacts of large herbivores have changed has been difficult to assess because direct measurements of their past impacts are rare, in part because faunal and vegetation records are often not found in the same depositional environments (Gill 2014). Moreover, causally linking herbivores to ecological change has been challenging, as temporal uncertainties in the palaeoecological record make it hard to determine whether the extinction of large herbivores preceded ecological change or vice versa (Gill 2014).

Given the constraints of the palaeoecological record, complementary approaches are necessary when exploring how these herbivore losses influenced ecological processes globally. An alternative method to estimate herbivore impacts indirectly is by studying the functional traits of herbivores. Functional traits determine how species interact with their environment and can be used to make inferences about species' ecological impacts (Malaterre et al. 2019). In conjunction with species distribution data, functional trait data can be used to model the distribution, form and intensity of herbivore impacts (Hempson et al. 2015). This approach can be used to functionally compare herbivore assemblages and their theorized impacts across time and space, even when these assemblages possess different species compositions (Gill 2015; Hempson et al. 2015). This makes the framework well‐suited to study problems where direct measurements of herbivore impacts are impossible.

Here, we estimated how much the late‐Quaternary extinctions have changed herbivore impacts by comparing the functional composition of current large mammalian herbivore assemblages to those of a no‐extinction scenario (so‐called present–natural assemblages, see methods). If herbivore losses did, indeed, cause major ecological changes throughout the late‐Quaternary, we would expect to observe major differences in herbivore functional composition, reflecting major changes in herbivore impact (e.g., see Karp et al. 2021). Conversely, if herbivore functional changes were small, they are unlikely to have been the driving force behind these late‐Quaternary ecological changes.

Next, we compare these differences in functional composition to those between contemporary ‘herbivomes’. Herbivomes are regions with a distinct form and intensity of herbivory, analogous to the biome classifications used in vegetation ecology (Hempson et al. 2015). We use the functional differences between contemporary herbivomes to delineate where herbivore assemblages have changed so much they can be considered functionally novel (Kerr et al. 2023). We consider assemblages functionally novel if they have changed so much that they would have been classified as a different herbivome.

Finally, we overlay our map of novel herbivore functional compositions with the ‘uncertain ecosystems’ map of Bond (2005). Uncertain ecosystems are ecosystems where the biome state depends on the disturbance regime present, such as herbivore impacts, rather than on climatic controls. By overlaying these maps, we highlighted regions that (1) have experienced large changes in herbivore impact and (2) where the vegetation should be sensitive to changes in herbivore impact. As such, we estimate where novel herbivore compositions would have had the largest consequences on vegetation states.

## 2 Materials and Methods

### 2.1 Modelling Herbivore Assemblages

To model the functional composition of assemblages, we classified herbivores (*n* = 502, *n*_extant_ = 302, *n*_extinct_ = 200) into functional types (species with similar functional effect traits and hence similar ecological impacts) (Malaterre et al. 2019; Table 1), using life habit, body mass and diet information for all wild large mammalian herbivores that existed during the last 130,000 years (i.e., the period covering all late‐Quaternary extinctions). We collected trait information from the HerbiTraits v1.1 dataset (Lundgren et al. 2021), which is partially based on trait information from PHYLACINE v1.2 (Faurby et al. 2018) and the Mass of Mammals dataset (Smith et al. 2003). We removed species lacking range maps in the International Union for the Conservation of Nature (IUCN) Red List (the primate *Piliocolobus pennantii* and artiodactyl *Gazella marica*).

**TABLE 1:** The list of herbivore functional groups. The table lists the name of each herbivore functional group, the percentage of habitable cells occupied by each functional group in the present–natural scenario, the percentage of habitable cells occupied by each functional group in the current scenario, the number of species belonging to each functional group (extant and extinct), and an example species for each group.

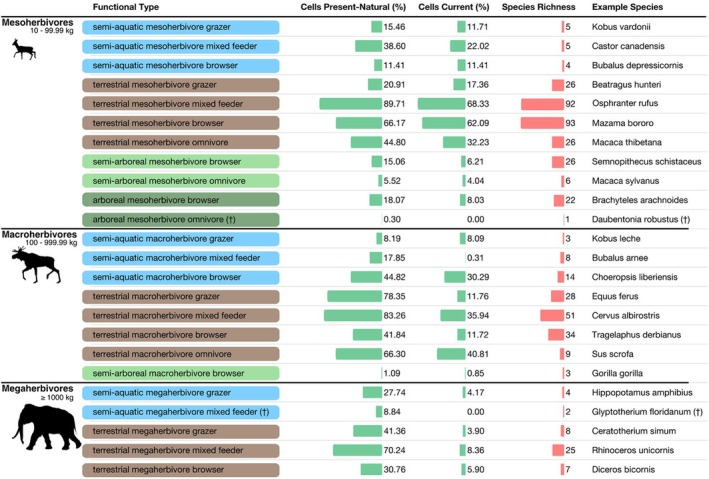

The trait data of extinct species are more uncertain than those of extant species and often come from different sources. Lundgren et al. (2021) base the trait data of extinct species primarily on trait values measured and reported in the scientific literature. Life habit data were also inferred from species' ecology. If no measurements were available, they were imputed. In the case of missing mass values (6 species in our dataset), they relied on the phylogenetic imputations from PHYLACINE v1.2.1. In the case of missing diet values (26 species in our dataset), the authors imputed the traits themselves. Both imputations were conducted using 1000 mammal phylogenies from PHYLACINE v1.2.1. For detailed information on how the traits were collected or imputed, we refer to Lundgren et al. (2021) and Faurby et al. (2018).

We assigned all remaining herbivores to a functional type by making classes of unique trait combinations. First, we classified herbivores into mesoherbivores (10–99.99 kg), macroherbivores (100–999.99 kg) and megaherbivores (≥ 1000 kg) following the body mass classification by Owen‐Smith (2013). Each body size group was then subdivided based on species' life habit classifications (semi‐aquatic, terrestrial, semi‐arboreal or arboreal). Last, we divided each of the resulting subgroups based on species' diet classification (grazer, mixed‐feeder, browser, omnivore). This gave us 24 different functional types. It is conservative to use such broad categories when estimating change as it removes some of the uniqueness of species and increases the likelihood of detecting similarities between assemblages. Moreover, extinct species lacking precise trait data can still be classified when using broad functional categories.

We collected species' current and present–natural ranges from the PHYLACINE v1.2.1 dataset. Current ranges are species' present‐day ranges, as defined by the IUCN Red List. Present–natural ranges are estimates of where species would occur today if their ranges had not been affected by humans during the last 130,000 years and while also accounting for environmental changes that have occurred during that time (Faurby et al. 2018). For most extant species, their present–natural range is identical to their IUCN range. Yet, if species experienced human‐caused range modifications, the present–natural ranges predict the distribution without anthropogenic modifications. For extinct species, the present–natural ranges range is estimated using a variety of methods, but in most cases, it is based on the present‐day occurrence of species with which the extinct species coexisted (Faurby et al. 2018). All ranges were analysed as rasters using the Behrmann equal‐area projection (96.5 × 96.5 km resolution at 30° N and 30° S).

We assumed that all species co‐occurring within a raster cell formed an assemblage and constructed herbivore assemblages by overlaying species ranges. We constructed current assemblages by overlaying current ranges, and we constructed present–natural assemblages by overlaying present–natural ranges. We defined the functional composition of herbivore assemblages as the relative proportion of functional types present. We did so by counting the number of species belonging to a functional type and dividing that number by the total number of species in the assemblage. The resulting relative richness values add up to one when all values within an assemblage are summed. As species belonging to similar functional types have similar ecological impacts, herbivore assemblages with similar functional compositions should possess comparable herbivore impacts (Hempson et al. 2015).

### 2.2 Visualising Functional Change

**FIGURE 1:**
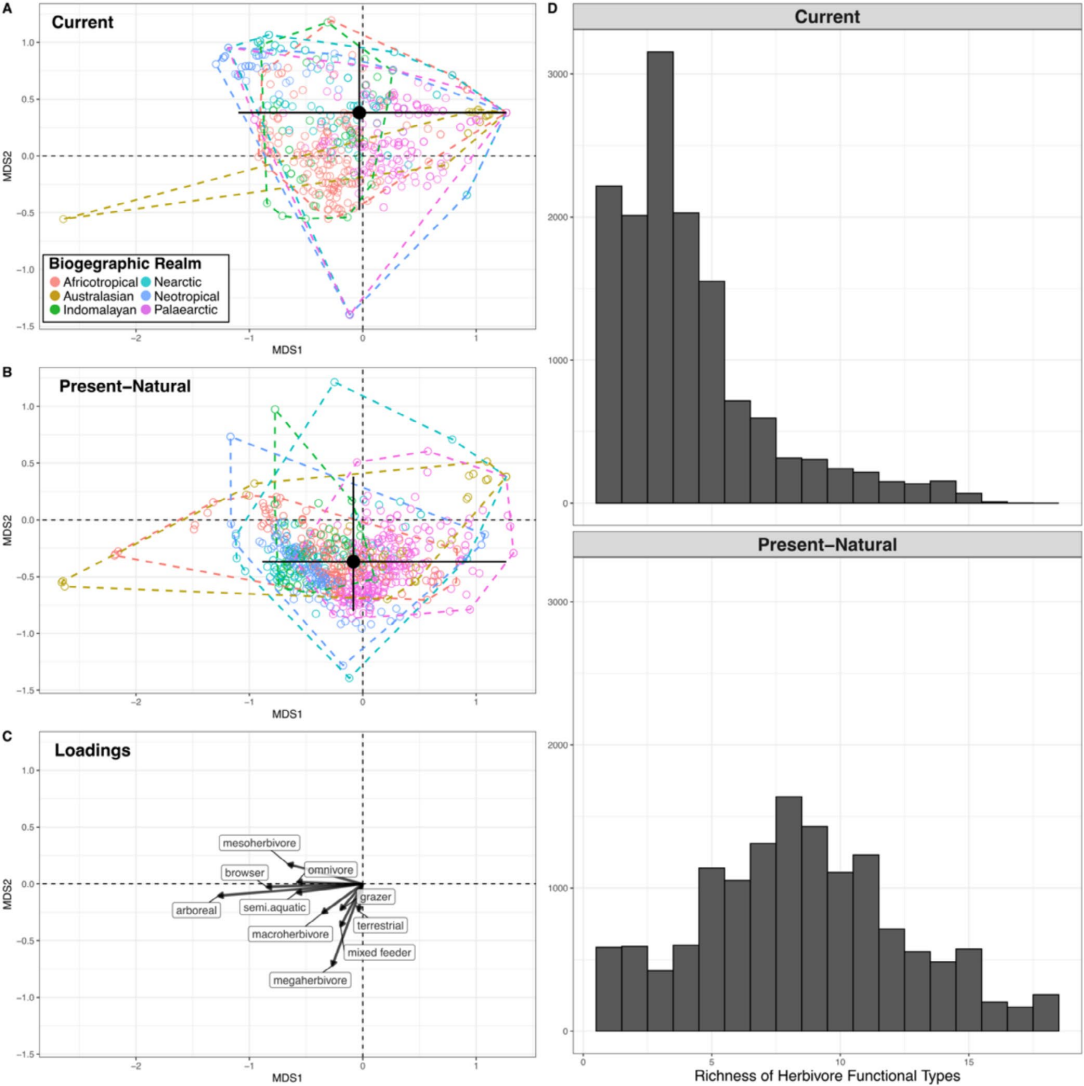
(A) 500 randomly selected current herbivore assemblages plotted in compositional space. (B) 500 randomly selected present–natural herbivore assemblages plotted in compositional space. The black dot denotes the median position of all assemblages. The black crossmarks indicate the range in which 95% of all assemblages are located. (C) The loadings of the Multidimensional Scaling. For visualisation, we have averaged the loadings to reflect traits, rather than the 24 functional types. (D) Histograms showing the functional richness of all current and present–natural herbivore assemblages.

To visualise differences between current and present–natural assemblages, we performed non‐metric multidimensional scaling on one thousand randomly selected herbivore assemblages based on the relative richness of each of the 24 functional types (500 current and 500 present–natural assemblages, reduced to three dimensions, Table S1). This gave us an ordination space where functionally similar assemblages clustered together (Figure 1).

In addition, we used the relative richness values to calculate the squared chord distance between assemblages. The squared chord distance is a distance metric commonly used in palynology that ranges from zero to two. A value of zero means both assemblages share all species (here, functional types), and that said species occur in the same proportions. A value of two means that the assemblages have no species in common. The squared chord distance is generally perceived as a good distance metric to assess assemblage similarity because it strikes a balance between up‐weighing rare taxa or types, and not being overly responsive to noise in the data (Jackson and Williams 2004). If cells possessed large herbivores in the present–natural situation but not in the current situation (1% of habitable cells), we could not calculate the squared chord distance. However, we assumed these current assemblages to be novel because they did not share any species, and we assigned them a squared chord distance of 2.

To delineate at what point compositional differences were so large that current herbivore assemblages could be considered functionally novel, we used the ‘herbivome’ classifications from Hempson and colleagues (Hempson et al. 2015). ‘Herbivomes’ are regions with a distinct form and intensity of herbivory, analogous to the biome classifications used in vegetation ecology. Herbivore assemblages belonging to different ‘herbivomes’ are distinct by definition. We, therefore, calculated the average squared chord distance between contemporary herbivomes (Figure S1, Table S2) and assumed that changed assemblages could be considered ‘novel’ if the difference between a current and a present–natural cell was equal to or larger than the average distance difference between present‐day herbivomes. Such a change would be functionally equivalent to an assemblage of African rainforest herbivores turning into an assemblage of savanna herbivores.

To calculate the dissimilarities between herbivomes, we digitised the herbivome maps in Hempson et al. (2015) using QGIS. We created polygons for all four herbivomes. However, we subtracted a 150 km buffer on the inside of the polygon to avoid digitisation errors, as they could lead to the inclusion of cells from other herbivomes. While we could visually discern the ‘Forest Duiker herbivome’ (a herbivome associated with tropical forests and non‐social browsers) and the ‘Arid Gazelle herbivome’ (a herbivome associated with arid regions and medium‐sized social mixed‐feeders) identified by Hempson and colleagues, we could not distinguish between the ‘bulk feeder’ herbivome and the ‘High VALS’ herbivome, both of which are associated with savannas. Consequently, we decided to lump the latter two herbivomes together. This gave us three herbivomes, instead of the initial four. These three herbivomes broadly corresponded to the major biomes of sub‐Saharan Africa: tropical rainforest, savanna and desert. Because our goal was to find a minimum threshold to determine novel assemblages, this method was the most conservative. If we had kept the ‘bulk feeder’ herbivome and the ‘High VALS’ herbivome as separate entities—even though our methods could not distinguish between them—their dissimilarity would be low. This would have given us a low threshold value, and could have caused us to classify assemblages as novel even though they are not very dissimilar.

**FIGURE 2:**
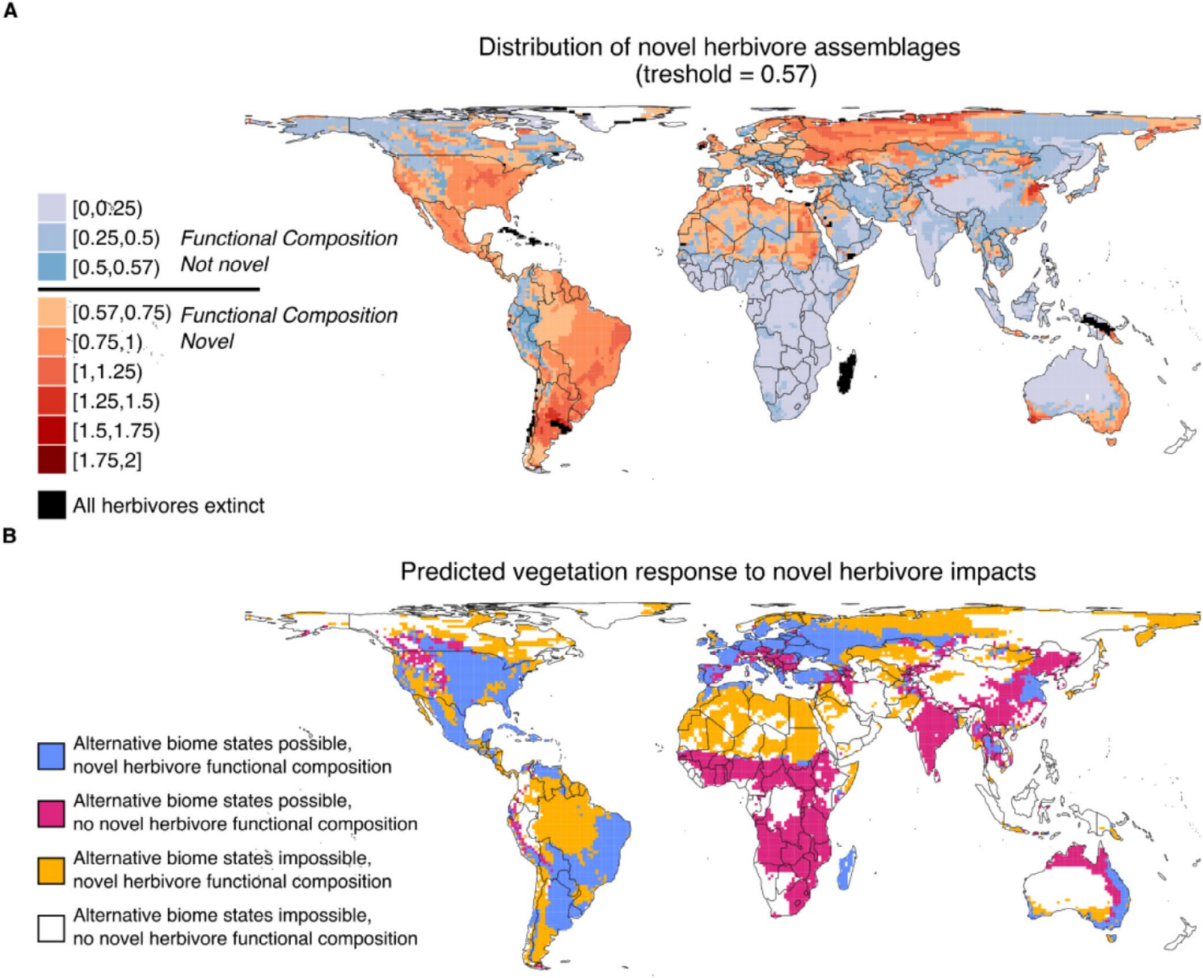
(A) The pair‐wise squared chord distance (ranging from 0 to 2) between current cells and the present–natural cell in the same location, a measure of their functional similarity. Cells are coloured blue if their dissimilarity values are below 0.57, meaning current assemblages are considered functionally similar to present–natural assemblages. Cells are coloured orange–red if their dissimilarity values exceeded 0.57, meaning current assemblages are considered functionally novel to present–natural assemblages. Black cells denote areas where all large herbivores have disappeared following extinctions. They are novel by definition. The values are binned in 0.25 steps, except around the threshold value of 0.57 where we constructed two unequal, smaller bins. (B) The predicted vegetation responses to novel herbivore functional compositions. We overlayed the areas where Bond (2005) predicts that alternative biome states are possible with our map of novel herbivore functional compositions. This divides the world into four different classes: (1) areas sensitive to changes in herbivore impact with novel herbivore functional compositions (blue), (2) areas sensitive to changes in herbivore impact without novel herbivore functional compositions (pink), (3) areas not sensitive to changes in herbivore impact with novel herbivore functional compositions (yellow), and (4) areas not sensitive to changes in herbivore impact without novel herbivore functional compositions (white).

We calculated the smallest average difference between present‐day herbivomes (0.57), and we used this value as a threshold to delineate whether assemblages were so functionally dissimilar as to be classified as ‘novel’. We calculated the squared chord distance between current assemblages and the present–natural assemblage that would occur in the same location, and we mapped the differences between them, using the threshold to indicate novelty (Figure 2A). We also considered a more liberal scenario (threshold = 0.4), based on squared distance values used in comparable vegetation studies, and a more conservative scenario using the second smallest average distance between herbivomes (threshold = 0.81) (see Supporting Information).

Finally, we overlaid our map of novel herbivore assemblages with the uncertain ecosystems map, defined by Bond (2005) (Figure 2B). We identified the areas where alternative biome states are possible according to Bond (2005) by selecting all cells that met the following two conditions: MAP > 7.143 × MAT + 286 and MAP < −1.469 × MAT^2^ + 81.665 ×MAT + 475 (MAP = mean annual precipitation; MAT = mean annual temperature). We collected annual temperature and precipitation data from WorldClim version 2.1 (Fick and Hijmans 2017).

## 3 Results

We identified 24 different functional types of herbivore, most of which were mesoherbivores (< 100 kg) and most of which had terrestrial lifestyles (Table 1). In addition, small‐bodied functional types were more species‐rich than large‐bodied functional types (i.e., more species would belong to that functional type). Notably, all but one herbivore functional type (semi‐aquatic mesoherbivore browser, e.g. *Bubalus depressicornis* ) had reduced ranges compared to the present–natural scenario. These range reductions were most pronounced amongst mega (≥ 1000 kg) and macroherbivore (100–999.99 kg) functional types (Table 1). Yet, despite range declines being common, only two functional types (arboreal mesoherbivore omnivores and semi‐aquatic megaherbivore mixed feeders) have become globally extinct.

### 3.1 How Are Current and Present–Natural Assemblages Different?

Calculating the squared chord distances between current and present–natural assemblages revealed that functionally changed herbivore assemblages are common; 94% of current assemblages were not functionally identical to their present–natural counterparts (squared chord distance > 0). Current and present–natural assemblages occupied distinct regions of compositional space (i.e., an ordination space where functionally similar assemblages cluster together). Moreover, current assemblages were less clustered around the centroid (Figure 1A,B), meaning they had, on average, less uniform functional compositions than present–natural assemblages. However, they also had more similar convex hulls, suggesting they contained fewer assemblages with outlying functional compositions. Current assemblages were characterised by an absence of large‐bodied functional types, a comparatively larger proportion of small‐bodied functional types (Figure 1C), and an overall lower richness of functional types than present–natural assemblages (Figure 1D).

### 3.2 Functionally Changed Herbivore Assemblages Are Widespread

In 47% of grid cells, the squared chord distance between current and present–natural assemblages exceeded the threshold for being functionally novel (Figure 2A). Novel assemblages occur in all biogeographical realms and cover large parts of the Nearctic, Neotropics and the Western Palaearctic. They are common on islands (e.g., Madagascar, the Caribbean and New Guinea, areas where all large herbivore species have gone extinct) and in the coastal areas of Australia. In contrast, current assemblages resembling the present–natural state remain prevalent in the Afrotropics, Indomalaya, Eastern Palaearctic and central Australia (Figure 2A). When we took a more liberal threshold of 0.4, in line with previous literature on plant communities (Jackson and Williams 2004), 64% of cells were identified as novel (Figure S2). When using a threshold of 0.81, the second smallest difference between herbivores, 23% of cells were identified as novel (Figure S3).

### 3.3 Where Would Changing Herbivore Impacts Have the Largest Influence?

We overlaid our map of functionally novel herbivore assemblages with the uncertain ecosystems map (Bond 2005) to identify areas (1) that had experienced large changes in herbivore impact and (2) where the vegetation should be sensitive to changes in herbivore impact. Such areas cover about 20% of terrestrial land, primarily across the western Palaearctic and the Americas (Figure 2B). The Afrotropical and Indomalayan regions, as well as Northern Australia, are also sensitive to changes in herbivore functional composition, but they have, so far, experienced comparatively few changes in the functional composition of their herbivore assemblages, also covering about 20% of terrestrial land. In about 27% of land, primarily in North Africa, the Amazon, the Arctic and southern South America, large changes in herbivore impact have occurred, but according to Bond (2005) the changes in disturbance regime should not lead to alternative vegetation types.

## 4 Discussion

We detected widespread functional differences between current and present–natural assemblages, indicating that the impacts of many wild herbivore assemblages (i.e., foraging behaviour, movement patterns, diet selection, etc.) have probably changed as a result of the late‐Quaternary extinctions. On almost half of the world's land, these functional differences were so stark that modern ecologists would classify corresponding current and present–natural assemblages as belonging to different herbivomes, regions with a distinct form and intensity of herbivory (Hempson et al. 2015). In other words, in large parts of the world, natural areas likely experience fundamentally different herbivore impacts than they would in the absence of the late‐Quaternary extinctions.

A growing number of scientific studies propose that herbivore extinction during the Late Pleistocene and Holocene has led to widespread changes in vegetation (Bakker et al. 2016; Gill 2014), fire regime regimes (Karp et al. 2021), vegetation consumption (Pedersen et al. 2023) and nutrient fluxes (Doughty et al. 2016; Smith, Hammond, et al. 2016). Our study models the theoretical impact of herbivores and does not show empirically how herbivore extinction led to ecosystem changes across the planet (i.e., changed vegetation dynamics, biomass removed, nutrients transported, etc.). However, our findings confirm that the functional changes in herbivore assemblages (and thus in impact) have been widespread and ecologically meaningful.

The functional differences we detected between current and present–natural assemblages mirror the global taxonomic richness declines that happened during the late Quaternary. They were least severe in Africa and Southeast Asia, both places with a long history of human presence. In contrast, they were more severe in Europe, North Africa, the Americas, parts of Australia and island ecosystems, often places where humans have more recent histories (Lemoine et al. 2023; Sandom et al. 2014). Moreover, just like with taxonomic extinctions, these functional changes were caused by the loss, rather than the replacement, of functional types.

The late‐Quaternary herbivore extinctions created functional voids that are evolutionarily unusual. For example, herbivores with large body sizes have been ubiquitous across terrestrial ecosystems for more than 50 million years, and their widespread absence in mainland ecosystems has not been seen since the early Cenozoic (Janis 2008; Smith et al. 2010; Svenning et al. 2024). Likewise, the low dietary diversity of current assemblages contrasts sharply with the composition of assemblages occurring during the last 10 million years, generally composed of herbivores with a diverse range of dietary strategies (Janis 2008; Schowanek et al. 2021). Recent studies suggest Neogene and Pleistocene megafauna assemblages displayed functional stability over periods of 700,000 years or longer, even though their taxonomic composition was variable (Cooke et al. 2022; Faith et al. 2019; Stegner and Holmes 2013). Functional downgrading by the late‐Quaternary extinctions thus appears to have disrupted certain forms of long‐term functional stability and may drive novel ecological and evolutionary dynamics globally, compared to mid‐ and late‐Cenozoic ecosystems (Cooke et al. 2022; Stegner and Holmes 2013).

### 4.1 Conservation Implications

Our findings highlight how much of the world's herbivore assemblages have been subject to significant reductions in functional richness. This accentuates how unique herbivore assemblages are that have retained high functional richness, such as in sub‐Saharan Africa and South‐East Asia. This is particularly the case for assemblages that have retained megaherbivores, as these functional types experienced some of the most severe range reductions, and their functional groups had the lowest species richness (meaning fewer species can replace their ecological impacts). Moreover, it is important to protect these assemblages, not only because they are functionally unique, but also because many of them occur in regions that may respond strongly to changes in herbivore impacts (Pausas and Bond 2020). Empirical evidence from herbivore‐controlled savannas in Africa provides an example of how the loss of large herbivores can lead to drastic vegetation changes in the landscape (Asner et al. 2009; Cromsigt and te Beest 2014).

In addition, our results suggest that bringing back extinct herbivores can play an important role in global restoration efforts, particularly if functional replacements are considered. Vegetation responses to herbivores depend on herbivores' functional traits rather than on notions of nativeness (Lundgren et al. 2024), and only two of our functional groups have disappeared globally, despite widespread taxonomic extinctions. As such, there may be several opportunities for functional restoration with functional substitutes. That said, our study uses broad trait classifications and likely overestimates the functional similarity between species (Daskin et al. 2023). Detailed ecological research is, therefore, needed to determine to what extent non‐native herbivores can replace the role of extinct herbivores.

Finally, our findings show how changes in herbivore impact cannot be fully understood without knowledge of the ecosystem in which they occur. As proposed by Bond (2005), in some ecosystems, changes in herbivore impact do not lead to biome changes (though herbivores can still have important ecological effects, e.g., Berzaghi et al. 2019). Yet, in other ecosystems, where alternative vegetation states are possible, similar changes in herbivore impacts could have drastic effects. Our results particularly highlight the Afrotropics and Indomalaya as relatively intact regions where alternative vegetation types are possible. These regions could thus be severely impacted if the remaining herbivore species go extinct. Conversely, if reintroductions can reverse the effect of earlier species losses (Alston et al. 2019), much of Europe and North America could see large vegetation changes following herbivore comebacks. The vegetation in much of these regions is sensitive to the disturbance regime but contains functionally simplified herbivore assemblages. Finally, our results could also help to identify where evidence of past herbivore‐induced vegetation changes should be easiest to detect, which may help palaeoecological research make sense of contradicting findings (e.g., Barnosky et al. (2016)). Nevertheless, more empirical work is needed, particularly outside of Africa, to confirm that these theoretical predictions happen on the ground.

### 4.2 Limitations

There is inherently some uncertainty surrounding the traits of extinct species and the functional composition of present–natural assemblages. First, trait data from extinct species tend to have higher uncertainties than those of extant species, as these cannot be measured on living specimens. This uncertainty could inflate the number of extinct functional groups. The extinct functional groups we identified both contain few species. Consequently, if the constituent species' traits turn out to be incorrect, these functional groups could easily cease to exist. A possible example is the extinct group of ‘semi‐aquatic megaherbivore mixed feeders’. It is composed of two related species that are classified as ‘semi‐aquatic’ in Lundgren et al. (2021), though, more recent studies suggest they may have been terrestrial instead (Fariña et al. 2013).

Second, there are biogeographical differences in data quality and completeness, which could influence the accuracy of the present–natural maps. Famously, there are more palaeoecological records from North America and Europe than from other regions (Nanglu and Cullen 2023). As such, our findings are more certain in the Nearctic and the Western Palaearctic. Likewise, the absence of change—particularly in the Southern hemisphere—could reflect a lack of data, rather than stable conditions.

Importantly, though, the present–natural assemblages used in our study are not past assemblages. They are counterfactual estimates of species richness that estimate what macroscale species richness patterns would be like today if late‐Quaternary extinctions had not occurred (Faurby et al. 2018). Many of the extinct species' present–natural ranges are estimated based on the ranges of extant species with which the extinct species co‐occurred at fossil sites. Even with well‐dated remains, some time averaging at fossil sites is likely: species that did not occur together in life will co‐occur as fossils in the same deposit (Behrensmeyer et al. 2000). Theoretically, this could give rise to some combinations of species that never co‐occurred, inflating the similarity between present–natural assemblages. That said, we do not think the time averaging of fossils can completely explain the increased similarity of present–natural assemblages. Present–natural ranges for extinct species were generally based on the co‐occurrence with multiple surviving species (Faurby et al. 2018). Moreover, many megafauna are known to co‐occur and interact, and several lines of evidence suggest that the co‐occurrence of mammals was higher during the Late Pleistocene (Smith, Tomé, et al. 2016; Tóth et al. 2019). We also note that the current assemblages are, likewise, based on coarse range maps that may overestimate species co‐occurrences and inflate assemblage similarities.

Despite these uncertainties, we believe our estimates are conservative. First, we quantified changes in the functional composition of herbivore assemblages, irrespective of changes in herbivore density. Incorporating density would increase the likelihood of finding novel assemblages, as wild herbivore densities have declined considerably during the late Quaternary (Manzano et al. 2023). Second, we use (idealised) range maps to estimate species presence‐absences. Yet, range maps often overestimate species distributions and do not take into account that many herbivores are locally absent or rare inside their range and, therefore, functionally extinct. In fact, much habitable land has been converted for agriculture and contains few wild herbivores even if range maps suggest they are locally present. A comparison that realistically incorporates human land use and its effect on wild herbivore densities will likely find even larger differences. Third, we consider assemblages novel only if their functional differences are at least as large as those observed between herbivomes in sub‐Saharan Africa today (squared chord distance = 0.57). This is a stringent standard, as continental‐scale studies have used Squared Chord Distances of as much as 0.4 to delineate novel (plant) assemblages, and most studies at intermediate scales use values ranging from 0.12 to 0.20 (Jackson and Williams 2004). Finally, and most importantly, our classifications of herbivore functional groups are broad. If even broad classifications fail to find functional similarities between current and present–natural assemblages, finer classification will only capture more functional differences. We, therefore, suspect that our findings underestimate, rather than overestimate, the degree of functional changes that have occurred.

## 5 Conclusion

We detected widespread functional differences between current and present–natural large herbivore assemblages, indicating that the impacts of many wild herbivore assemblages have changed following the late‐Quaternary extinction wave. Many terrestrial, natural areas across the planet now experience fundamentally different disturbance regimes than they did before the late‐Quaternary megafauna extinctions. While more empirical work is needed to show how these functional changes in herbivore assemblages have affected ecosystems, our findings suggest that they have been widespread and ecologically important. Scientific research and restoration efforts should pay more attention to the evolutionarily unusual and simplified nature of most present‐day large herbivore assemblages and the implications for ecosystem functioning and biodiversity.

## Author Contributions

**Simon D. Schowanek:** conceptualization (equal), data curation (equal), formal analysis (equal), visualization (equal), writing – original draft (equal), writing – review and editing (equal). **Matt Davis:** conceptualization (equal), supervision (equal), writing – review and editing (equal). **Erick J. Lundgren:** conceptualization (equal), data curation (equal), writing – review and editing (equal). **Owen Middleton:** conceptualization (equal), data curation (equal), writing – review and editing (equal). **John Rowan:** conceptualization (equal), data curation (equal), writing – review and editing (equal). **Daniel Ramp:** writing – review and editing (equal). **Christopher J. Sandom:** writing – review and editing (equal). **Jens‐Christian Svenning:** conceptualization (equal), funding acquisition (equal), supervision (equal), writing – review and editing (equal).

## Conflicts of Interest

The authors declare no conflicts of interest.

## Supporting information


Data S1.

## Data Availability

Scripts and data can be accessed on the lead author's Github page.
