# Supplementary material for: The Late‐Quaternary Extinctions Gave Rise to Functionally Novel Herbivore Assemblages
Source: Ecol Evol. 2025 Mar 13;15(3):e71101. doi: 10.1002/ece3.71101 (PMC11906253; doi:10.1002/ece3.71101)
Supplement: Supplementary file 1 — Data S1. [file ECE3-15-e71101-s001.docx]

# Supplementary Information

## NMDS Loadings

| *Table S1: NMDS Loadings*  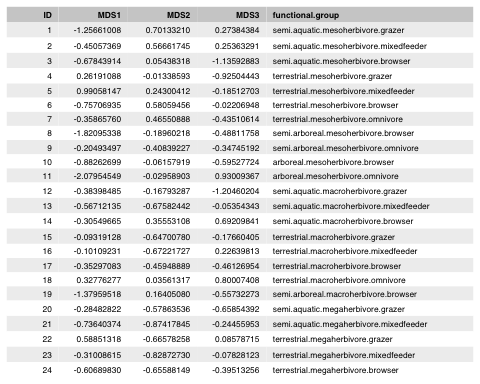 |
| --- |

## Differences Between Herbivomes

| 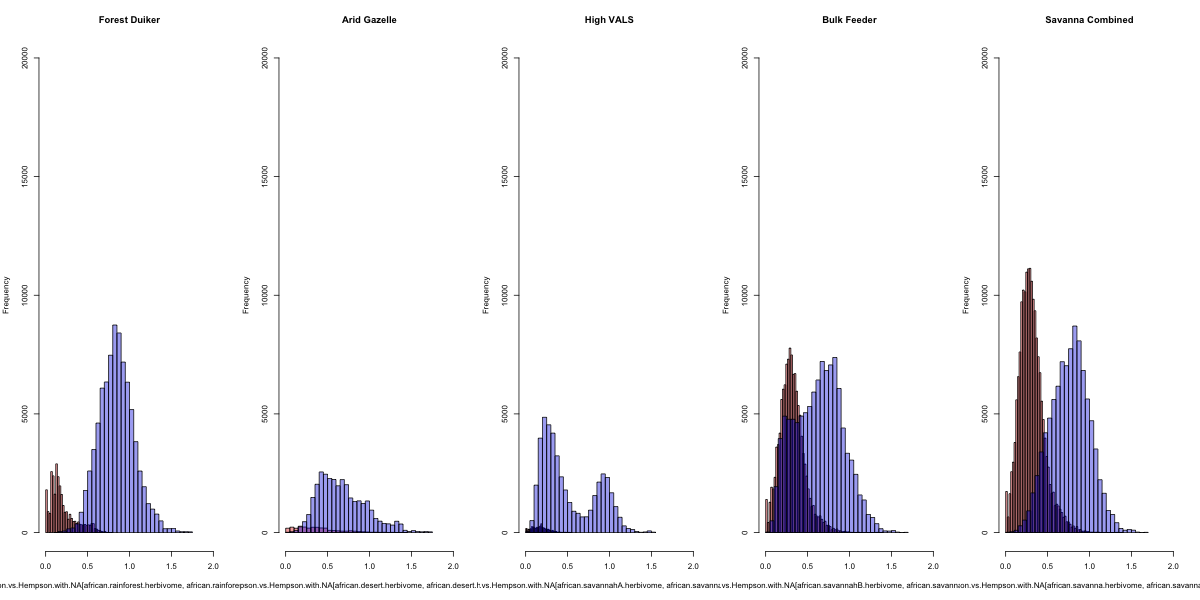  *Figure S1: The distances between cells belonging to the same and different herbivomes.* |
| --- |

| *Table S2: The average distance between assemblages belonging to the same and different herbivomes.*  X between within ratio 1 Forest Duiker 0.85 0.14 6.07 2 Arid Gazelle 0.57 0.26 2.19 3 High VALS 0.36 0.16 2.25 4 Bulk Feeder 0.72 0.30 2.40 5 Savanna Combined 0.81 0.28 2.89 |
| --- |

## Maps

### Liberal Estimates

| 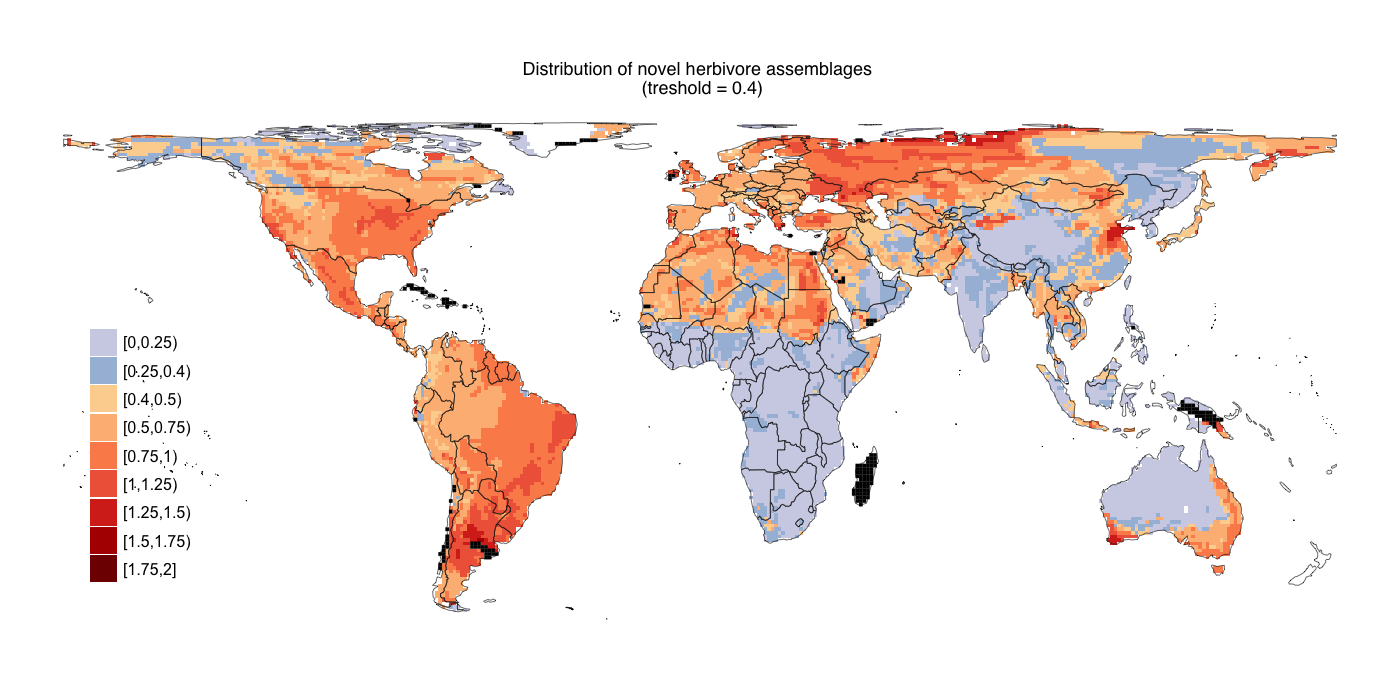  *Figure S2: The pair-wise squared chord distance (ranging from 0 – 2) between current cells and the present-natural cell in the same location, a measure of their functional similarity. Cells are coloured blue if their dissimilarity values are below 0.4, meaning current assemblages are considered functionally analog to present-natural assemblages. Cells are coloured orange-red if their dissimilarity values exceeded 0.4, meaning current assemblages are considered functionally novel to present-natural assemblages. Black cells denote areas where all large herbivores have disappeared following extinctions. They are novel by definition. The values are binned in 0.25 steps, except around the threshold value of 0.4 where we constructed two unequal, smaller bins.* |
| --- |

### Conservative Estimate

| 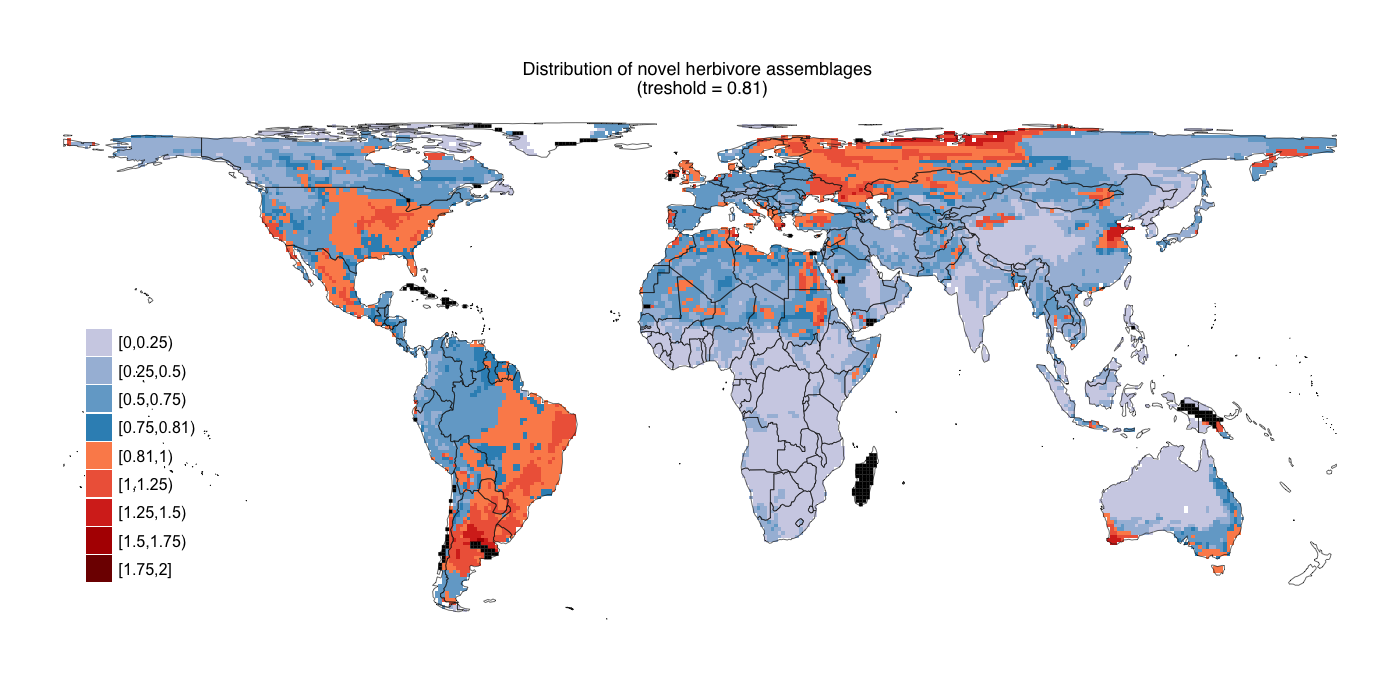  *Figure S3: The pair-wise squared chord distance (ranging from 0 – 2) between current cells and the present-natural cell in the same location, a measure of their functional similarity. Cells are coloured blue if their dissimilarity values are below 0.81, meaning current assemblages are considered functionally analog to present-natural assemblages. Cells are coloured orange-red if their dissimilarity values exceeded 0.81, meaning current assemblages are considered functionally novel to present-natural assemblages. Black cells denote areas where all large herbivores have disappeared following extinctions. They are novel by definition. The values are binned in 0.25 steps, except around the threshold value of 0.81 where we constructed two unequal, smaller bins.* |
| --- |
